# The ethics and logistics of field-based genetic paternity studies

**DOI:** 10.1017/ehs.2020.23

**Published:** 2020-05-13

**Authors:** Brooke A. Scelza, Elizabeth G. Atkinson, Sean Prall, Richard McElreath, Jacob Sheehama, Brenna M. Henn

**Affiliations:** 1 Center for Behavior, Evolution and Culture, UCLA, Los Angeles, CA 90095, USA; 2 Department of Anthropology, UCLA, Los Angeles, CA 90095, USA; 3 Broad Institute, Harvard University, Cambridge, MA 02142, USA; 4 Interdepartmental Doctoral Program in Anthropological Sciences, SUNY Stony Brook, NY 11794, USA; 5 Department of Anthropology, University of Missouri, Columbia, MO 65211, USA; 6 Department of Human Behavior, Evolution and Culture, Max Planck Institute for Evolutionary Anthropology, Leipzig 04103, Germany; 7 Medical Biochemistry and Microbiology, University of Namibia, Oshakati, Namibia; 8 Department of Ecology and Evolution, SUNY Stony Brook, NY 11794, USA; 9 Department of Anthropology, UC Davis, Davis, CA 95616, USA

**Keywords:** anthropological genetics, paternity, ethics, methods

## Abstract

The rapidly decreasing costs of generating genetic data sequencing and the ease of new DNA collection technologies have opened up new opportunities for anthropologists to conduct field-based genetic studies. An exciting aspect of this work comes from linking genetic data with the kinds of individual-level traits evolutionary anthropologists often rely on, such as those collected in long-term demographic and ethnographic studies. However, combining these two types of data raises a host of ethical questions related to the collection, analysis and reporting of such data. Here we address this conundrum by examining one particular case, the collection and analysis of paternity data. We are particularly interested in the logistics and ethics involved in genetic paternity testing in the localized settings where anthropologists often work. We discuss the particular issues related to paternity testing in these settings, including consent and disclosure, consideration of local identity and beliefs and developing a process of continued community engagement. We then present a case study of our own research in Namibia, where we developed a multi-tiered strategy for consent and community engagement, built around a double-blind procedure for data collection, analysis and reporting.

## Media summary

Paternity testing in anthropology raises ethical and methodological issues. We summarize these and describe a novel double-blind method used in our work.

## Introduction

One of the distinguishing characteristics of anthropological genetics is its focus on remote and geographically diverse populations (Crawford, 2000, 2007). That emphasis has been key to work on understanding genetic diversity and phenotypic plasticity, both of which are hallmarks of the field. Where genetic sampling has been combined with ethnographic and demographic data we have gained an even greater understanding of population history and dynamics (Destro-Bisol et al., 2004; Fix, 1978; Gopalan et al., 2017; Ober et al., 1997; J. Turner, 2013; Wen et al., 2004; Wilkins & Marlowe, 2006; Yashin et al., 1999). However, most of this integrative work has, to date, relied on previously collected demographic databases, ethnohistorical data or summary statistics that demonstrate variation across cultures. Much less common is the pairing of detailed, individual-level anthropological data with genetic sampling. Such studies have the potential to be transformative to the field. For example, understanding not only the means but also the variance in the timing of life history events would aid studies of population divergence by providing more accurate measures of generation time (Moorjani et al., 2016; S divergence by pr14). Similarly, more detailed data on birthplace would aid microevolutionary work on patterns of genetic diversity across geographic space, as well as work on migration rates (Verdu et al., 2010). Sexual and marital histories could be used along with genetic data to better understand reproductive skew and the links between mating success and reproductive success, similar to work done in other species (Altmann et al., 1996; Say, Naulty, & Hayden, 2003; Widdig et al., 2004).

While there are myriad benefits to these integrative studies, they bring with them a host of ethical and logistical challenges. Data collected during health and life history interviews are often sensitive and confidential. Furthermore, studies of kinship routinely address both biology and social norms, potentially putting the evidence that comes from genetic testing in opposition to locally accepted ideas. The incongruity of these two kinds of data raises important questions about the primacy of scientific knowledge and the determinants of identity, kinship and history (Royal et al., 2010; TallBear, 2013).

One area where these issues are particularly acute is genetic paternity studies. Biological paternity is, by its nature, obscured, Anthropologists studying kinship and demography must rely on individual assertions, or short of that generalized cultural norms, to understand patterns of parentage. Geneticists, on the other hand, can easily determine biological paternity, providing a concrete answer to an otherwise convoluted question, but their data may not conform to local kinship beliefs. Here, we share our experience working with a community of Himba agro-pastoralists to develop and implement a method for combining genetic paternity data with detailed demographic and ethnographic interviews (Scelza, Prall, & Starkweather, 2020a). Our aim is to not to offer a one-sized fits all method for this type of work, but rather to suggest some baseline recommendations for developing studies that integrate genetics with sensitive personalized data like paternity and provide one method for addressing these issues.

### The relevance of paternity to anthropology and genetics

Among mammals, humans have relatively high levels of paternal investment (Clutton-Brock, 1991; Geary 2000). At the same time, women in many societies regularly engage in concurrent partnerships (Broude, 1980; Scelza, 2013a). The coupling of these two features leads to a significant conundrum for men: how to accurately direct investment toward their biological kin (Alexander, 1974). Attempts to address this problem reach into many areas of anthropological inquiry. For example, there are tight links between inheritance patterns and female fidelity, with matrilineality occurring predominantly in societies with low paternity certainty (Hartung, 1985). Similarly, kinship terminology differs across societies in accordance with the level of paternal uncertainty (Greene, 1978). Where resources are passed down through the patriline, there is often strict control of female autonomy. A desire to curb female infidelity has been linked to a number of cultural practices including veiling, purdah, foot-binding and female genital mutilation (Wilson & Daly, 1995). The broad influence that paternity has on cultural and family systems highlights the importance of having a better understanding of actual rates of nonpaternity across cultures.

In the absence of genetic paternity data, anthropologists have for the most part relied on generalized norms about infidelity as a cue to paternity certainty and tend to construct broad hypotheses about how paternity will affect behavior (e.g. paternal relatives will invest less than maternal ones) (Gaulin, McBurney, & Brakeman-Wartell, 1997). A few studies have tied measures of paternity confidence to measures of investment or bias (Alvergne, Faurie, & Raymond, 2009; Anderson, Kaplan, & Lancaster, 2007; Apicella & Marlowe, 2004), but having access to genetic paternity data would open the door to a range of new and exciting questions. For example, how accurate are men and women in their paternity assertions? What mechanisms provide the best cues to genetic parentage and what countermeasures are most effective in obscuring paternity? How do nonpaternity events impact reproductive skew? How effective are different types of social norms, religious systems or mate guarding behaviors in limiting extra-pair paternity?

Understanding concurrency from a female perspective has also been of longstanding interest to biological anthropologists. Contrary to traditional views of multiple mating (*sensu* Bateman), which posit only limited benefit to females, women can gain from partnering with multiple men, either sequentially or concurrently (Hrdy, 2000; Scelza, 2013a). For example, among Bari and Ache foragers, having multiple named ‘fathers’ of a child, assigned through cultural systems of partible paternity, is beneficial to women and children, with children who have more than one father more likely to survive than children with a single father (Beckerman et al., 1998; Hill & Hurtado, 1996). Concurrent partnerships may provide women and their children with greater resource security or abundance, in addition to other social benefits (Walker, Flinn, & Hill, 2010).

The combination of demographic, ethnographic and genetic data would open up many new ways to study how female concurrency affects reproductive and health outcomes. For example, does genetic similarity to one’s marital partner predict the likelihood that she will have children fathered by other men? This might be particularly important in small populations where overall homozygosity is higher. Does the level of genetic similarity (e.g. across the Human Leukocyte Androgen (HLA) system) between women and their marital partners predict the likelihood that she will have extra-marital partners or extra-marital children? Does it predict length of marriage or other measures of marital satisfaction? Data on the importance of HLA to partner choice and partner satisfaction vary widely (Havlicek & Roberts, 2009; Winternitz, Abbate, Huchard, Havlíček, & Garamszegi, 2017), but rarely do studies have long-term demographic data or ethnographic context to draw from.

Despite its relevance to a wide range of issues within both anthropology and genetics, studies of nonpaternity in humans continue to be quite rare. Many existing studies use data from paternity clinics, which represent a biased sample of the population (Anderson, 2006). Among studies of European and American populations, rates of nonpaternity are currently believed to be quite low, between 1 and 5% in a given generation (Larmuseau, Matthijs, & Wenseleers, 2016). However, there is almost no paternity data from nonwestern countries (for an exception see Strassmann et al., 2012) and given the cross-cultural range in the frequency of concurrent partnerships, more studies are clearly needed. However, addressing these questions requires more than just running paternity tests in a wider range of populations. It entails linking genetic results with individualized data on household demography, beliefs, preferences and investment patterns. It also requires the kind of detailed understanding of local context that comes through long-term ethnographic study.

### Introduction to the case study

Our aim in this paper is to use our experience implementing a study of marital and family dynamics as a conduit for exploring the ethical and logistical issues associated with genetic paternity studies more generally. We begin by introducing the population with whom we work and providing some general context about the project, before delving into specific issues of project design, implementation and evaluation.

The Kunene Rural Health and Demography Project was initiated in 2010, with a focus on understanding how family and marital dynamics contribute to reproductive decision-making. The project is co-directed by two US-based researchers (Scelza and Prall) and a researcher from the University of Namibia (Sheehama). Ethnographic work and demographic interviews in the first years of the project revealed a consistent pattern of concurrent partnerships by both men and women, raising intriguing questions about the role of extra-pair paternity in this population. However, the genetic component of this project is just one among many methods and topics the project is focused on. Using ethnographic, demographic and health data we have published studies addressing jealousy (Scelza, 2013b), partner choice (Scelza & Prall, 2018), maternal and child health (Prall & Scelza, 2017; Scelza & Hinde, 2019), sleep (Prall, Yetish, Scelza, & Siegel, 2018), marriage and inheritance patterns (Scelza, 2011; Scelza, Prall, & Levine, 2019) and parenting decisions (Scelza et al., 2020a; Scelza & Silk, 2014).

The project is longitudinal in nature, and concentrates on 40–45 households, with whom we have regular contact. At least one member of the project goes to Namibia each year, along with a team of Namibian research assistants, many of whom have worked multiple field seasons. This means that we have now had the opportunity to work with many members of the community for years, interviewing them on multiple occasions and developing a considerable level of trust and rapport.

We began the process of integrating genetic data into the project in 2013 with a site visit by Dr Brenna Henn. Dr Henn was chosen both because she had expertise with African genetic samples and because she had previously conducted significant amounts of fieldwork in Southern Africa. Initial conversations were held between Scelza, Henn and local chiefs and heads of household about the possibility of genetic testing, a description of how saliva samples could reveal information about ancestry and paternity, and a discussion of how this work would fit in with our larger project on marital and family dynamics. At that time we began discussions about how disclosure would be handled, in particular the risks involved with revealing nonpaternity events. Permission was granted to conduct pilot testing, which began the following year. In the interim, the research team designed a double-blind method for conducting analyses, which fit with the desires of the community, as expressed during our initial meetings (described below). Data collection continued over the next few years. This slower pace proved to be advantageous in that it ensured that there were plenty of opportunities to continue discussions with the community and to become reassured that the consent procedure and double-blind process were well understood. Data cleaning, extraction and analysis were then completed in the US, according to the double-blind protocol outlined below. Results were then presented to the community. Upon discussion and approval of the community to proceed with dissemination, the research team then presented the results to scientific communities in Namibia and the US and proceeded with publication.

### Himba beliefs about fatherhood and paternity

In order to understand how we framed and developed our study of paternity, and its relation to other aspects of our work, an introduction to the ways that Himba men and women think about partnerships, parenting and paternity is needed.

The basic notion that Himba and (closely related) Herero commonly have both marital and non-marital partners has been written about in ethnographies from throughout the twentieth century (Gibson 1958; Harpending and Pennington 1990). These older ethnographies describe the practice as being not only common, but also carrying minimal sanctions. Authors also note that distinctions between the social father (*pater*) and the biological one (*genitor*) are made and highly relevant to thinking about partnerships and paternity, with agreement among ethnographers that social fatherhood is of primary significance. Gibson writes, ‘One Herero baldly declared that it made no difference whether other men came to sleep with his wives, for the children would belong to him regardless’ (Gibson 1958, p. 28).

These observations fit with the demographic data BS began collecting in 2010. In a first round of interviews with reproductive- and post-reproductive-aged women, interlocutors volunteered information about the assignment of paternity to social vs. biological fathers, noting that certain children were from their husbands, while others were fathered by ‘lovers’. It was only after these assertions came up voluntarily that we began systematically collecting these data. The following year, interviews with men commenced, and they too spoke freely about which children born to their wives they believed they were the biological father of, and they also provided the number of children they believed they had fathered with women outside of their marriages.

Throughout, in both semi-structured interviews and informal conversations, the notion of the importance of social fatherhood was central. Men spoke about their role and responsibilities as the father of children born to their wives, which exists even when they are certain that they are not the biological father. For example, marriages between pre-pubertal girls and men are common, although the act is ceremonial and not consummated. Later, when the girl reaches menarche, the marriage may be consummated and the couple will begin to co-reside. However, in about a third of these cases, the bride never goes to live with the husband and the marriage is never consummated. In these cases, if that young woman goes on to have children before marrying again, her husband from the child marriage is the social father of those children. Those are cases where he can be certain he is not the biological father, and yet he maintains obligations to help care for those children. Understanding Himba norms and practices related to this dual nature of fatherhood, and being able to link DNA results with these demographic and ethnographic data was critical to interpreting the genetic data that we collected.

## Designing field-based paternity studies

There are a host of ethical challenges associated with paternity testing even in clinical settings where clients are literate, individuals have a basic knowledge of genetics and one or both parties has initiated the testing themselves (Rothstein, 2005). The large literature on the ethics of paternity testing has focused mainly on clinical settings in western or urban communities, and tends to concentrate on two types of ethical conundrums. The first are cases where nonpaternity is revealed incidentally, typically when genetic testing is done for another reason. The second are cases where either the mother or the putative father initiates genetic testing in order to conclusively determine paternity. In either circumstance, there can be emotional, material and legal ramifications that stem from a revelation of nonpaternity. In response to this, ethicists and clinicians have discussed whether and to whom nonpaternity should be revealed (Hercher & Jamal, 2016; Lucassen & Parker, 2001; Ross, 1996; Wright et al., 2002), how paternity data should be used in the courts (Draper & Ives, 2009) and how paternity testing complicates notions of fatherhood (Kaebnick, 2004).

While these discussions have made important contributions that can guide anthropologists in their work, field-based paternity studies must engage with a somewhat orthogonal set of issues. As opposed to a clinical setting, paternity testing in anthropological research typically takes place inside communities, in conjunction with other kinds of engagement such as interviews or participant observation, and ideally in the context of longer and deeper relationships between those conducting the test and those being tested. In addition, it is typically the researcher seeking the paternity result, rather than the parties being tested. To address these differences, we will focus on three areas of study design and execution that we propose should be of primary importance when developing an ethically sound protocol for paternity testing in the field: (a) informed consent and disclosure decisions; (b) consideration of local identities, beliefs and biases; and (c) continued community engagement. Following this, we introduce the double-blind procedure for paternity testing we developed, which allows for individual-level analyses while posing minimal risk to participants.

### Informed consent

The process of how best to obtain informed consent continues to be debated in both academic and policy circles. Most recently, revisions to the Common Rule have set forth updated requirements for obtaining informed consent that focus on including a reasonable and understandable presentation of information (Office for Human Research Protections, 2017). It also requires that researchers be clear about confidentiality and whether or not de-identified data can be used for future studies without requiring re-consent. These elements of the rule are particularly relevant to anthropologists, who often work with vulnerable populations. Low literacy levels, language barriers, collective decision-making norms and group ownership of ‘genetic property’ all pose challenges that anthropologists may contend with when they are developing a consent process (Dodson & Williamson, 1999; Marshall, 2003). Added to this is the fact that genetic studies pose problems because they entail explaining abstract scientific concepts (e.g. what is a ‘gene’?) and drawing links between the biological sample (saliva or blood) and the information of use to the researcher (DNA). This raises issues about ‘readability’ and ‘true understanding’ in consent forms that the new rule was designed to address (Hodge & Gostin, 2017; Menikoff, Kaneshiro, & Pritchard, 2017), but which have been the subject of discussions about consent in the social sciences for more than a decade (Marshall et al., 2006; Paasche-Orlow, Taylor, & Brancati, 2003). There already exist some excellent examples of how to tackle these issues in populations with low rates of education and nonwestern cultural practices (Bhutta, 2004; Rotimi & Marshall, 2010).

One key difference between anthropological studies and clinical studies is that anthropological studies typically involve whole communities, rather than isolated individuals who often make up clinical samples. This difference should be reflected in the consent process, which can benefit from community-level engagement and discussion that precedes individual consents (Ramsay et al., 2014). Involving the community in the development of the consent process itself has several advantages. It allows paternity researchers and the communities in which they work to discuss the pros and cons of disclosure (see below) and other relevant issues before testing begins. These conversations provide opportunities for the community to learn about genetic testing, to provide input about locally relevant barriers to consent and to make a mutually agreed upon plan for data use and sharing.

Anthropologists can learn from some of the methods being developed in biomedical research to update and improve methods for informed consent. Rather than thinking of consent as a static document, or providing ‘broad consent’ for wide-ranging data use, new methods encourage ‘dynamic consent’ (Budin-Ljonsentoura., 2017; Kaye et al., 2015; Steinsbekk, Myskja, & Solberg, 2013). Dynamic consent allows participants to have ongoing communication with the research team and control over the use of their data, and is said to improve trust between researchers and participants, as well as allow participants to have a more active role in the research process (Stein & Terry, 2013). A limitation to dynamic consent is that it requires that participants have the ability to continually access information about the study (typically done online), which may be difficult or impossible in some places. However, with increasingly globalized communication and access to cell phones and email, mechanisms like text messages could be employed. To our knowledge, dynamic consent has not been formally implemented in an anthropological genetics study, but biological anthropologists have advocated for greater consideration of its use, and we add our voices to that call (Turner, Wagner, & Cabana, 2018).

In our study consent and community engagement were intimately linked. Development of the consent was informed by years of previous ethnographic research, as well as conversations with community members specific to this project. In addition, the slow process of data collection allowed time for conversations between the pilot and main data collection, which ensured that community members understood the consent and disclosure process. For example, if we had had many individuals asking us for individual results after the pilot testing, that would have been a sign that people did not understand (or remember) that these results would be blinded (see below for details on double-blind procedure). Similarly, if people were asking us during other interviews to reveal paternity results, that would be a sign that they did not know about the double-blinding. In fact, we had only one case of someone asking for an individual result in the following years, and this person was quickly reminded of the double-blind procedure, without further incident.

Our consent explicitly addressed current and future use of the data, as well as where the genetic data would be stored. We discussed use of the data to trace ancestry at a broad level (e.g. the relationship of Himba to other ethnic groups) as well as at the familial level (parent–child relationships). The consent reads, ‘DNA tells us about your ancestors and about how you are related to other Himba in this community. It will tell us how children are related to parents. We will also be able to learn about things like how many of the children in this community are “omoka”‘. We then go on to explain the double-blind aspect of the procedure including that we would not be reporting individual-level data back, and what information we, the researchers, would have access to. We also explicitly addressed future use of the data, stating that their genetic information would not be sold, but that it would be shared with other researchers and that the genetic information would be deposited in a database shared with scientists from around the world. Genetic information from this project has been deposited in the Database of Genotypes and Phenotypes, which is managed by the National Center for Biotechnology Information. There is no phenotypic information included. Pedigree relationships in the database are constructed only from the genetic data (information that is encoded in the genome), so that other researchers cannot determine rates of extra-pair paternity as they do not have information on social relationships or marriages.

### Disclosure decisions

In the clinical literature, there has been robust debate about whether and to whom paternity results should be disclosed (Bellis, Hughes, Hughes, & Ashton, 2005; Lucassen & Parker, 2001; Zabidi-Hussin, 2012). Most of this discussion has centered on cases where nonpaternity is revealed incidentally while testing for another condition. On the side of disclosure, the determination of various committees, as well as large surveys of geneticists, has been to reveal results to the mother, but not to the putative father in order to preserve families and minimize conflict (Pencarinha, Bell, Edwards, & Best, 1992; Wertz, 1999). Others have gone further, arguing for full disclosure to both parties. The arguments in favor of full disclosure include respecting the autonomy of individuals and their ability to make informed choices and discouraging clinicians and researchers from being unnecessarily paternalistic (Lucassen & Parker, 2001; Ross, 1996).

While a partial or full disclosure policy may work well in clinical settings, there are both practical and ethical reasons to question such a practice in anthropological research (Marshall & Koenig, 2004). Sharing genetic paternity information with only a subset of the community, such as with mothers only, may be viewed as unfair, and at times may be wholly impractical owing to communal governance or gender hierarchies. Even if formal permission for this type of reporting was allowed, there could be repercussions down the line, as women may be pressured to reveal what they know. Furthermore, because the nature of anthropological work is that it involves intimate relationships between researchers and interlocutors, there may also be direct pressure on anthropologists to reveal paternity results informally (e.g. ‘just between you and me, is he my son?’).

In response to some of these concerns, some researchers have advocated for universal nondisclosure (Mandava, Millum, & Berkman, 2015; Palmor & Fiester, 2014). Several case studies that considered the familial and social ramifications of disclosure concluded that the risks of revealing misattributed paternity outweighed the costs of withholding that information (Adlan & Have, 2012; Avci, 2017). A 2015 Hastings Center Report, which argues for universal nondisclosure, offers some practical suggestions for developing consent forms that minimize the costs of withholding paternity results (Mandava et al., 2015). First, to counter the notion that withholding paternity results could lead individuals to believe their social father is in fact their biological father, the authors suggest a transparent consent process that clearly states that paternity results will not be reported back. The second issue relates to betrayals of trust, where individuals feel it is the obligation of the researcher, especially where there are longstanding relationships, to disclose important personal information. The authors again advocate for a clear consent process stating that such information will not be disclosed. One solution is to inform participants about how they can seek paternity results on their own (Palmor & Fiester, 2014), although this might be impractical in more remote settings.

Ultimately, it is up to the research team and the community with whom they work to decide whether disclosure is appropriate, and if it is opted for, how to integrate it into the overall communication plan for the research study. In our own work (details below) we opted for a double-blind system, where neither the anthropologists nor the community would receive individual-level results, but aggregate results would be presented back.

### Risks and benefits

One important aspect of consent relates to the disclosure of risks and benefits. Because paternity is so tightly linked to decisions about investment, revealing genetic results that conflict with an individual’s own assessment of paternity could have a direct effect on how men provide material or emotional support in the future. Revealing a nonpaternity event could also trigger marital dissolution or spousal abuse (Bellis et al., 2005). Because of this, discussion of the risks of paternity studies have centered mainly on the repercussions of data sharing rather than on any risks inherent in the data collection process iteself (Gutmann et al., 2012).

There are distinct sets of considerations and risks associated with paternity studies in groups that have purported high and low rates of extra-pair paternity. Where extra-pair paternity is expected to be rare, there are often strong norms and laws in place that punish individuals for infidelity. In such cases, revelations of nonpaternity can put people at significant risk of social stigma, emotional trauma and/or physical danger. While we would expect societies that strongly discourage extra-marital relations would also have low rates of extra-pair paternity, there will almost always be individual cases of nonpaternity uncovered during data analysis (Anderson, 2006).

Somewhat counter intuitively, it is the places where purported nonpaternity is low that the risks of revealing nonpaternity may be the greatest. This is true even if only aggregate results will be reported back because where extra-pair paternity rates are low and the study population is small, it may be fairly easy for community members to make plausible deductions about which children resulted from affairs. In some cases these risks may be significant enough that it is not ethical to conduct paternity tests in that population. In addition, cultures where the risk of extra-pair paternity is high tend to also be places where there are norms in place that support concurrent partnerships and de-stigmatize infidelity (Scelza, Prall, & Levine, 2019). While this can result in a reduction of risk within the group itself, revealing a high rate of extra-pair paternity could increase bias or stigma against that group by outsiders. In a review focused on genomic studies of disease, deVries and colleagues highlighted potential ways that stigma could be magnified by linking the susceptibility of disease with particular ethnic groups, importantly noting that this could then lead to discriminatory practices (de Vries et al., 2012). Similar considerations are relevant to paternity studies, where stigma about sexual practices could cause comparable harm. The same study showed that in cases where traditional and genomic histories are discordant, community members have been more likely to report harm than when the two narratives support one another.

These risks must be weighed against the gains in knowledge that can come from challenging conventional wisdom about sexual stereotypes. If paternity studies are limited to populations where the expected nonpaternity rate is low, notions of infidelity as ‘deviant’ are supported, which may not only be a misrepresentation of the true diversity in sexual practices, but could itself enhance stigma. In an evaluation of the group-level stigma that can come from genomic studies, it was argued that the risks will be highest in communities where the genetic study is the first to highlight a bias, and in groups where the trait being studied is already locally stigmatized (de Vries et al., 2012). It is up to the research team to weigh the more diffuse benefits of knowledge enhancement with the specific community-level risks of group harm. We acknowledge that striking an adequate balance on this front may not be possible in many places, which should lead researchers to seriously reconsider whether a study in such a population is warranted.

Given these risks, and that, like many types of basic research, the results of paternity studies are unlikely to be of direct benefit to participants, there is a strong imperative for researchers to think about other ways in which their work in a community can be of value. For anthropologists, this is often already an integral part of their work and can include providing basic health care, providing communal meals, donating supplies to a local school or community center, or providing transportation to local clinics, funerals or other events. Instead of providing payments for genetic testing in cash, researchers can think about providing other items that might be difficult to acquire and highly valued (e.g. reading glasses, solar chargers or cell phone recharge vouchers). These may be particularly useful if genetic studies are conducted alongside demographic or other interviews that take up a significant amount of time and require greater compensation. Longer-term, multi-year projects typically mean that researchers have a greater sense of the needs of the community and open up possibilities for working on other kinds of projects. Long-term work also allows researchers to bring back photographs taken on previous trips or present slideshows. Our work has followed this last model. While the community was open to our studying family and marital dynamics, there was no concrete benefit that came to them from this kind of basic science work. However, we have increasingly integrated measures of health and well-being into our study (e.g. child anthropometrics, food security, female autonomy), which will allow us to understand the impacts of long-term drought and stochastic resource access on people’s well-being.

The basic science approach that we have taken in our research has always been complemented by an effort to support the community while we are present. Participants were provided with food and small household items (e.g. washing powder) in exchange for their time. We also cook meals at our camp, which are open to whomever is around, and we regularly have 6–12 Himba eating with us. Our vehicles are also used to bring people to the clinic, to town, or to funerals or ceremonies when needed. In order to increase our ability to support the community year-round, we have also begun working with a nonprofit that has teamed up with the Namibian Red Cross to provide school supplies and new water pumps for communities in the area.

### Consideration of local identities, beliefs and biases

The paternity testing literature has raised important issues about the meaning of fatherhood (Kaebnick, 2004). Genetic tests threaten to distill parent–child relationships to their biological roots, and where these tests are given legal precedence this can affect both the emotional and material well-being of both parents and their children. This is particularly true in cases where either the mother uses paternity testing to try to obtain support or where fathers use the tests to evade these responsibilities. Children face their own dilemmas, in deciding whether and how much contact they want with their social and biological fathers when paternity results reveal a discrepancy. These issues are at the forefront in societies where biomedical beliefs about reproduction are predominant (Rothstein, 2005), but in many areas of the world the lines between social and biological fatherhood are more opaque. If there are not locally distinct concepts of social and biological fathers in a particular place, researchers need to seriously consider the impacts of revealing such information. For example, in many South American societies there is a predominant belief in ‘partible paternity’, where children are thought to have multiple biological fathers (Beckerman et al., 1998; Beckerman & Valentine, 2002). Paternity tests that provide a definitive and singular result would necessarily challenge these local beliefs.

A more common concern than the case of partible paternity relates to local norms about maintaining multiple partners. Where concurrency is common it is critical that researchers have a clear understanding of how concepts of marriage and parenthood are understood. For example, in our own work with Himba pastoralists concurrent relationships are very common and there is widespread acknowledgement that children are regularly born to men other than a woman’s husband. However, the concept of ‘social fatherhood’ is very strong, and there are norms in place promoting men’s investment in children born to their wives, regardless of whether they are the biological father (Scelza et al. 2020b; Prall and Scelza, 2020). Having an emic understanding of fatherhood in this population both helped us to frame discussions about paternity testing in culturally appropriate ways and ensured that we could engage with our results in more nuanced ways than if we had only the paternity results to rely on.

### Continued community engagement

It is increasingly common for anthropologists and geneticists to be expected to report results back to local entities (Bankoff & Perry, 2016; Tindana et al., 2015, 2017), echoing broader calls in both anthropology and biomedical research to increase transparency and collaboration in research (Fluehr-Lobban, 2008; Macauley et al., 1998; Turner et al., 2018; Weijer & Emanuel, 2000). This creates a considerable conundrum for anthropologists: how to conduct sensitive types of genetic testing like paternity analyses in ways that will protect the privacy of participants, while still allowing for open communication between researchers and the communities in which they work.

One way to address this concern is to develop an open and multi-tiered communication strategy. In a review of community engagement strategies, Tindana and colleagues found that these kinds of community engagement are valuable for two reasons (Tindana et al., 2015). First, beginning conversations early and incorporating local ideas and opinions led to greater ability for researchers to clear up misunderstandings about the project and to adapt procedural and consent materials to reflect local concerns and ideologies. Second, a continuing engagement allows for a more flexible process that can more closely mimic the kind of decision-making that occurs in small-scale settings, and be responsive to changing needs and concerns. Techniques like focus groups might be useful, particularly early on, in order for researchers to gain an understanding of relevant issues (e.g. concerns related to collection of bodily fluids, how to word sensitive questions) (Marsh et al., 2010; Tindana et al., 2012). In the context of longitudinal projects, which anthropologists often engage in, developing a more formal and permanent structure, like a community advisory board, might be useful and appropriate (Morin et al., 2008; Rotimi et al., 2007). Finally, just as paternity results are not collected in isolation, discussions of paternity testing should be discussed in relation to the other data (e.g. interviews, census or health data) that will be used in the study.

Mirroring the call for a multi-tiered communication strategy, consents can also occur at various levels. This could begin with engagement and information aimed at community leaders or councils, before individual-level testing begins (Rotimi et al., 2007). Depending on the context, permission to work within a household might also be required. Finally, individual-level consents would occur. Engagement and permissions from these various levels (either formally or informally) are key to developing a culturally sensitive research program (Jewkes & Murcott, 1996; Seeley, Kengeya-Kayondo, & Mulder, 1992).

Researchers should also plan return trips to the field for dissemination of results. The process and forum for dissemination should be worked out with the community, and might include presentations at community meetings or town halls, or more dispersed visits to households in places where large gatherings are rare or difficult. Whatever strategy is decided upon, it is critical that researchers build in support for that plan, including requesting funds for pilot or follow-up trips (Tindana et al., 2015). Finally, the timing of dissemination should also be considered. Presenting results back to the community before their publication allows for an additional opportunity for community members to engage with the study, provide context for the results and provide a final post-hoc form of consent.

In addition to engaging with focal communities, researchers should also be thinking about how to engage with the research community within the country where the research takes place. Unlike public health and biomedical scientists, anthropologists often work alone, which has precluded collaborations in the past. However, collaborative work is increasingly common, particularly in biocultural anthropology. These collaborations can ensure that local stakeholders in the academic community are able to inform the work, and to gain opportunities to participate and share expertise. The H3Africa project gone a long way toward modeling how to form collaborations between African and non-African researchers that contribute to capacity building and a reduction of the inequities that have historically plagued anthropological and genetics research (de Vries et al., 2015). Some principles which have been recommended by African researchers are to ensure that all parties are involved in the research design from the ground up (including possibilities for African researchers to design and conduct their own research) and that there are fruitful ways to exchange information throughout, resulting in long-term partnerships rather than short-term collaborations that end when data collection is complete (Munung, Mayosi, & de Vries, 2017). Where genetics are only a part of a larger, anthropological project, these kinds of engagements could include involving local researchers in other parts of the work, including those related to public health, ecology and environment, or demography.

Our process of community engagement began years before genetic testing commenced. Because BS had been working in the community since 2010, conducting demographic interviews and studying family dynamics more generally, the community was already aware of our interests in these topics. When we added the genetic component to our work, we did so in a slow and deliberate manner. We began with conversations with the chiefs about DNA and what could be measured. We discussed ancestry at a broad scale (i.e. population history) as well as at the level of families, directly highlighting the possibility that we would be able to determine genetic paternity. We then spoke with them about different possibilities for disclosure, and together came to the decision to withhold individual-level results from both our team and the community (see double-blind method description below). Instead we presented community-level aggregate results (percentage of extra-pair paternity children in the sample and confidence rates for men and women).

Because Himba had notions of social and biological fatherhood that mapped well onto biology, we were not in a position to counter folk-biological beliefs. We did, however, take great care to emphasize that we understood the distinction, and that we were not privileging biological paternity over social fatherhood. We have conducted several studies looking at these distinctions, and have had extensive interviews (formal and informal) with men about their roles as fathers (Scelza et al., 2020b; Prall and Scelza, forthcoming). Even so, there was a bias in people’s reporting of paternity assertions that reflected the importance they place on social fatherhood. Our genetic results reveal that while men are more than 70% accurate at detecting extra-pair paternity events, when they err, they do so almost exclusively in one direction: stating that a child is their biological offspring when it is not (Scelza et al. 2020a). When we discussed this finding with the community (before publication of the results), we were told that it was likely that people were in fact much more accurate than our data show, but that men (and women) would be likely to report to us that children were theirs, even if they believed them not to be, to make sure we understood that those children ‘counted for them’. We included this information in the paper reporting these results, both to be transparent about reporting bias, and to highlight the importance of the emic notion of social fatherhood.

**Figure 1.**
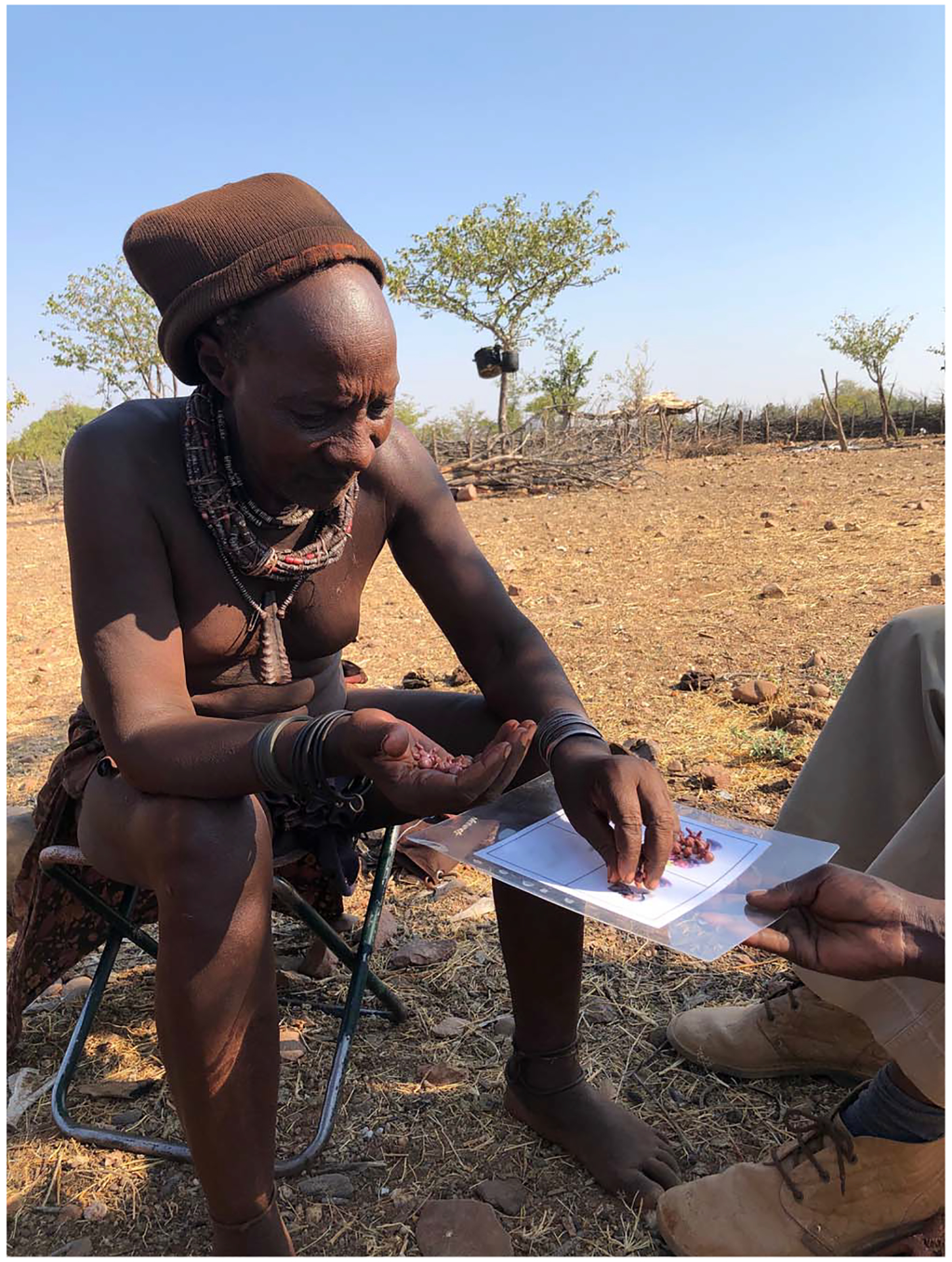
Himba man participating in task to assess community perceptions of the aggregate nonpaternity rate.

As much as possible, we tried to remain open to change during the research process. This included continual discussions with other anthropologists, economists and public health workers about our project. These insights allowed us to make adjustments when needed. In the most profound case, after describing the method to an economist at a small meeting, she noted that presenting the aggregate results back could itself be an intervention, if that aggregate result differed greatly from the community’s perception of the aggregate. Admittedly, this was not something that we had originally thought of. While we were relatively sure that our numbers would be unsurprising to the community (and in fact they were) owing to years of interviews with people on the topic, we wanted to know this for sure. We designed a simple task to run before presenting the results back, to assess the community aggregate (Figure 1). We had ~20 community members respond to the following: ‘If a typical Himba man had 10 kids, how many of them do you think would be from him, and how many from someone else’. We were fortunate that our ethnographic intuitions proved correct, and the community aggregate matched very well with our actual number. However, the prospect that they would not raised serious questions about what we would have done. We take from this first, the importance of conducting paternity studies only after having a deep ethnographic understanding of the topic in the community of interest, and second the value of developing tests like the one we conducted *before* data collection begins and having a plan (developed with the community) for how to proceed in the case of a mismatch.

Finally, we have attempted to use our project as a way to build bridges with the Namibian research community. Our collaborator comes from the field of Public Health, but as paternity has strong links to maternal and child health, STI transmission and (potentially) intimate partner violence, we felt this was a pertinent link. Our collaborator has visited the field site, advised us on procedures, and facilitated opportunities to share our results with Namibian colleagues. This culminated in a research conference held at the University of Namibia in 2018, and another presentation in 2019. This conference and the conversations that ensued from it have opened the door to collaborations that go beyond the scope of the paternity project, and which we hope will last many years.

### Double-blind procedure

Of paramount importance when conducting paternity testing in the field is ensuring that the work does not negatively impact family relations or the emotional, physical or material well-being of those being tested. While there is no one method that will be ideal for all settings, in our own work we opted for nondisclosure. To address some of the limitations of nondisclosure, including creating a disparity between researcher and community knowledge and the possibility of researchers being pressured to disclose, we devised a double-blind procedure for testing and analysis. We first outline the steps of this procedure and then discuss the benefits and limitations of using a double-blind method.

Double-blind protocols are typically used when researchers are concerned about imparting bias into a study. They have been used in behavioral studies where a researcher may unconsciously influence participant responses (Hoffman, McCabe, & Smith, 1996; Phillips et al., 1999). Here both the participants and the fieldworkers would be blinded to individual-level paternity results. This creates parity in the information available to the community and the researchers, while minimizing the chance that genetic testing could influence future investment and relationship dynamics.

Blinding is a useful tool to enforce universal nondisclosure, but it does not, in and of itself, allow for the integration of genetic data with other individualized traits. In order to conduct analyses that incorporate the full suite of information that anthropologists have, the double-blind method must be conducted by a team, each member of which is blinded to certain kinds of data. Following this procedure, none of the parties would have access to both individualized data and paternity results, yet analyses would be able to maintain important links between individuals (Table 1).

**Figure 2.**
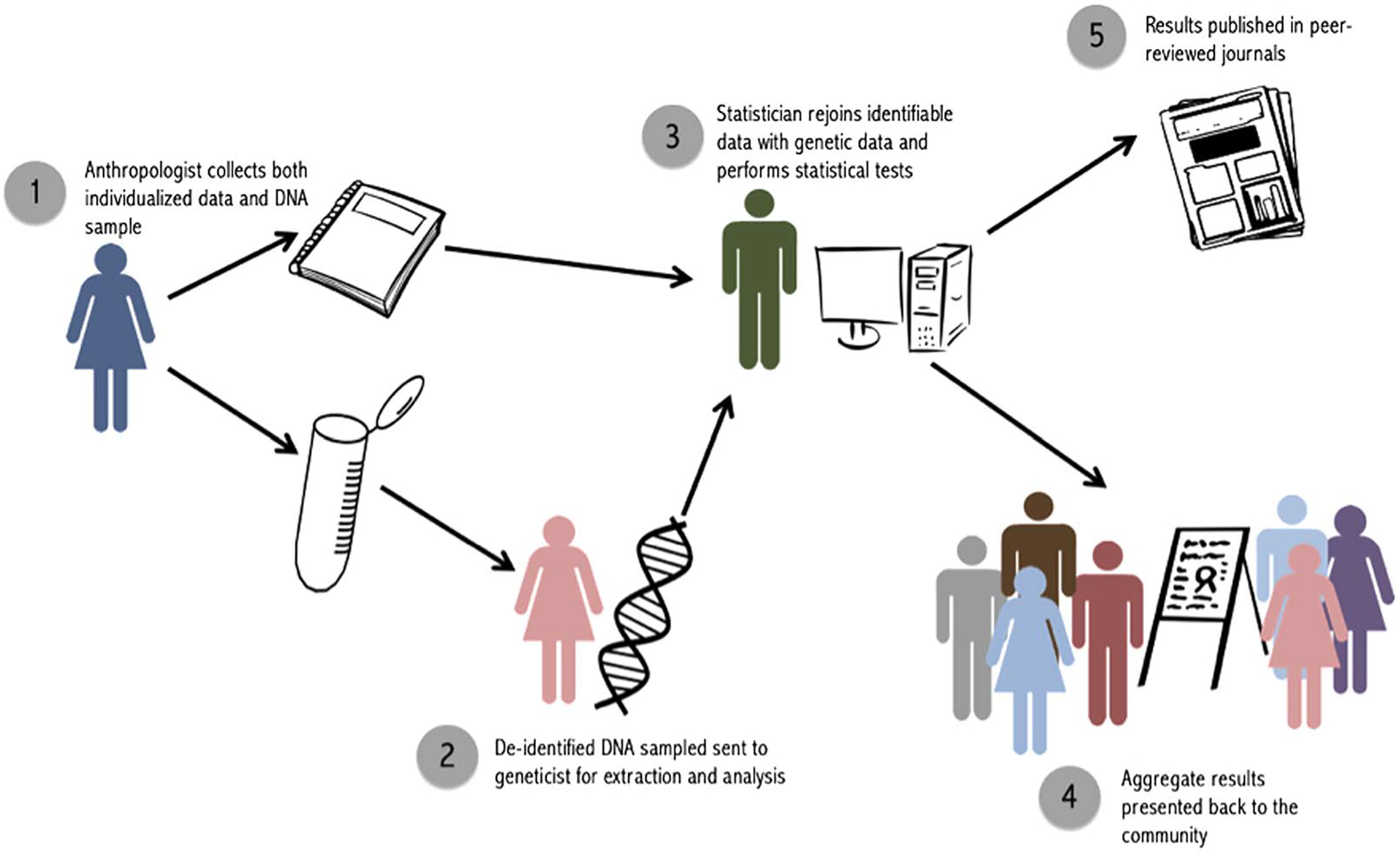
Steps to implement the double-blind procedure.

For our purposes, we label these three parties the ‘anthropologist’ the ‘geneticist’ and the ‘statistician’ (Figure 2). It is also possible to have only two parties involved, a fieldworker and a statistician, if genetic analyses are contracted out to an external laboratory and paternity results are sent directly to the statistician. The key is to ensure that the person who is collecting and maintaining identifiable data, and returning to the community to report back results, does not have access to individualized paternity results.

The ‘anthropologist’ is the key person in the field, in charge of collecting both genetic samples and individualized data. Ideally, this person will already have an extended relationship with the community, be aware of relevant cultural norms, and have familiarity with influential members of the community. It is this person, and this person only, who retains identifiable data linking an ID to attributes like names, dates and relationships to others in the study. Upon completion of fieldwork, the ‘anthropologist’ sends samples (labeled with ID number only) to the ‘geneticist’, who extracts DNA and performs paternity analyses. The ‘geneticist’ will thus have access to coded genetic relationships, family pedigrees and paternity results for particular dyads, but will not have access to any individualized information that could be used to identify participants. This person should not be involved in data collection or reporting to minimize the risk of accidental identification of individual paternity cases. However, as in our case, having the geneticist come to the fieldsite to participate in initial conversations with the community is helpful. Finally, the ‘statistician’ receives paternity results from the ‘geneticist’ and individual-level data (i.e. paternity assertions and an ID-coded pedigree) from the ‘anthropologist’. The ‘statistician’ uses IDs to link these results and calculate nonpaternity rates. The ‘anthropologist’ is returned the aggregate results of statistical analyses, but not any form of raw data that could be used to link results back to a particular person or dyad. The genetic data deposited into Database of Genotypes and Phenotypes includes only the transformed IDs available to the geneticist. Individual-level genetic results are not published, in accordance with our National Science Foundation data management plan, in order to protect the privacy and confidentiality of community members and abide by our double-blind procedure.

### Benefits of a double-blind procedure

First, for the community members involved, the price of revealing nonpaternity can be very high. It is not difficult to think of the negative ramifications that could occur when a father finds out that a child who he thought was his turns out not to be, including, but not limited to, withdrawal of investment, emotional dissonance, intimate partner violence and child abuse. While we recognize that the negative stigma attached to infidelity and nonpaternity varies cross-culturally, even where such practices are normative, there remains some potential for negative repercussion. Blinding the results, as part of a larger protocol for nondisclosure, helps to protect individuals from these kinds of harm.

Second, being blind to the results precludes those conducting fieldwork from unintentionally revealing results, either in casual conversation or through nonverbal tells. This is particularly important for anthropologists who return to the same small communities year after year.

Third, a double-blind method allows for transparent reporting because the fieldworker can reveal the entirety of what she knows (e.g. aggregate results), rather than withholding some information from the community.

### Limitations of a double-blind procedure

First, nondisclosure is an integral part of the double-blind procedure. As such, it may not be appropriate in all contexts, either when communities express a strong desire for transparency, or where the costs of nondisclosure are deemed to be greater than the risks of revealing nonpaternity.

Second, analyzing double-blind data can limit the available procedures for performing standard quality control on the genetic results. Sample swaps, either owing to mislabeling of DNA in the field or during extraction and plating in the wet laboratory, can be corrected by recollecting DNA from suspect samples or verifying ethnographic information during a second field season. However, as neither the geneticists nor the field anthropologist have access to both individual IDs and the paternity results, sample swaps become a more problematic feature of this design. One alternative correction is for the geneticists to check a wider set of kinships then merely the paternity assertion between dyads (e.g. siblings, grandparents). If an individual is an outlier for all reported family members, then the sample should be dropped as a labeling error.

Third, if anthropologists are blind to individual-level paternity results, they are unable to write about particular cases that might be interesting to highlight (e.g. if the chief has particularly high paternity certainty). While aggregate analyses allow for linking trait types with broad classes of paternity data (e.g. lower nonpaternity in love matches than in arranged marriages), it is often helpful for anthropologists to be able to map particular ethnographic data with specific results to help readers get a feel for why a particular pattern predominates. However, if genetic data is used to assess paternity confidence (the percentage of time that participants are correct in their assertions), and accuracy is high, researchers can discuss particular cases based on assertions, with the assumption that they are a reasonable proxy for genetic paternity. In addition, for certain measures like investment decisions, assertions may be more relevant than genetic paternity, as this is the currency people are actually evaluating when making their decisions.

## Conclusions

As the field of anthropological genetics continues to grow, so too do the benefits of collaboration between members of the field. One of the great advantages of these collaborations is the ability to utilize the wealth of individual-level data and the deep ethnographic and environmental knowledge anthropologists have about the places they work. The integration of these data with genetic sampling opens up whole new fields of inquiry. It also allows researchers to make more accurate assumptions in their theoretical and analytical models. For example, demographic interviews can be used to make more accurate estimates of generation time, which affect parameters like the germline mutation rate (Moorjani et al., 2016). Behavioral and other phenotypic data are also useful for elucidating specific hypotheses in gene association studies (Ilardo & Nielsen, 2018). For evolutionary anthropologists, there is a similar push to bring together proximate- and ultimate-level questions in more integrated studies (Nettle, Gibson, Lawson, & Sear, 2013). As our study shows, fostering these collaborations can benefit all parts of the research process, from the generation of research questions to the development of distinctly mixed-method studies, to the range of possible analyses. We therefore believe that further integration of genetic data with more traditional ethnographic methods will produce better and more ethically sound science.

## Figures and Tables

**Table 1.** Sources of data available to each member of the research team during a double-blind paternity study

	Anthropologist	Geneticist	Statistician
Participant identifiers	**×**		
Paternity results		×	×
Demographic/behavioral data	×		×
